# Peripheral sex differences in serum serotonin modulation in habitual short sleepers: an exploratory randomized trial of encapsulated *Lacticaseibacillus rhamnosus* GG

**DOI:** 10.1093/sleepadvances/zpag098

**Published:** 2026-09-02

**Authors:** Gonzalo Saiz-Gonzalo, Ahmed W Al-Humadi, Seamus McSweeney, Carel W le Roux, Sinéad B Bleiel

**Affiliations:** Anabio Technologies, Department of R&D, IDA Business Park, Carrigtwohill, T45 RW24, Cork, Ireland; Diabetes Complications Research Centre, Conway Institute, University College Dublin, D04V1W8, Dublin, Ireland; Anabio Technologies, Department of R&D, IDA Business Park, Carrigtwohill, T45 RW24, Cork, Ireland; Diabetes Complications Research Centre, Conway Institute, University College Dublin, D04V1W8, Dublin, Ireland; Diabetes Research Centre, Ulster University, BT52 1SA, Coleraine, United Kingdom; Anabio Technologies, Department of R&D, IDA Business Park, Carrigtwohill, T45 RW24, Cork, Ireland

**Keywords:** serotonin, sleep duration, gut-brain axis, *Lacticaseibacillus rhamnosus* GG, randomized trial

## Abstract

Sleep restriction, serotonin biology, and gut-brain signaling are interconnected, but whether habitual short sleep is associated with altered peripheral serotonin in healthy adults remains unclear. Probiotic modulation of circulating serotonin has also been insufficiently characterized. In this exploratory randomized, double-blind, placebo-controlled study, 21 healthy adults received microencapsulated *Lacticaseibacillus rhamnosus* GG or placebo for 28 days and were classified by habitual sleep duration as short sleepers (≤5 h/night) or adequate sleepers (≥6 h/night). Fasting serum serotonin was measured at baseline and day 28. Habitual short sleep was not associated with elevated serum serotonin. Female sex was associated with higher adjusted day-28 serum 5-HT in the primary winsorized log-transformed ANCOVA model (Type II ANOVA: F = 6.04, *p* = .03, partial η^2^ = 0.29), whereas treatment and sleep group did not show evidence of association. Although no statistically detectable independent probiotic effect was identified in this small exploratory sample. Directional decreases were observed in some participants with higher baseline serotonin values. These findings suggest that sex may be an important source of variation in peripheral serotonin and support further adequately powered studies using objective sleep phenotyping and prespecified sex-stratified analyses.

## Teaser

Habitual short sleep is common, but its relationship with peripheral serotonin in healthy adults is poorly defined. In this exploratory pilot trial, habitual short sleep was not associated with elevated serum serotonin, whereas sex emerged as a potentially important source of variation. Although no statistically detectable independent probiotic effect was detected in this exploratory sample, participants with higher serotonin baseline values showed directional declines within the microencapsulated probiotic arm. These findings support larger studies using objective sleep phenotyping, prespecified sex-stratified analyses, and standardized pre-analytical handling of peripheral serotonin.

## Introduction

Serotonin (5-hydroxytryptamine; 5-HT) is a signaling molecule involved in mood regulation, sleep–wake biology, and gut-brain communication. Although central serotonergic pathways are classically linked to vigilance and affect, most of the body’s 5-HT is synthesized peripherally, predominantly by enterochromaffin cells in the gastrointestinal tract. Gut-derived 5-HT enters the circulation and is avidly taken up by platelets; accordingly, peripheral measurements largely reflect a gut-platelet axis rather than direct brain serotonin biology [1–3]. This distinction is important when interpreting circulating 5-HT in human sleep studies.

Interest in peripheral 5-HT has increased with the recognition that the gut microbiota can regulate host serotonin biosynthesis. Experimental work showed that indigenous spore-forming bacteria promote enterochromaffin-cell 5-HT production and increase circulating platelet-associated serotonin, providing a mechanistic basis for microbiota-dependent modulation of peripheral serotonergic signaling [1]. Subsequent reviews have reinforced the concept that microbial products, enterochromaffin-cell responses, and platelet handling form an integrated pathway through which gut ecology may influence host physiology beyond the intestine [2, 3].

Sleep loss is another biologically plausible modifier of peripheral serotonin. In a controlled human metabolomics study, acute total sleep deprivation altered circulating serotonin-related signals, including serotonin and tryptophan, suggesting that short-term sleep loss can shift peripheral serotonergic biology [4]. However, findings from acute laboratory sleep deprivation cannot be assumed to generalize to habitual short sleep-in free-living adults, where chronic adaptation, behavioral heterogeneity, and environmental influences may yield a different physiological profile. At present, whether habitual short sleepers exhibit altered peripheral 5-HT remains unclear.

Sex may represent an additional, and often overlooked, source of variation in peripheral serotonin. Human data indicate that platelet serotonin uptake sites and serotonergic receptor binding vary across the menstrual cycle, supporting a role for sex hormones in platelet serotonergic handling [5]. This is relevant because serum 5-HT is strongly influenced by platelet sequestration and release during clotting, meaning that sex-related differences in platelet biology may materially affect measured concentrations. Accordingly, sex should be considered not merely as a covariate, but as a biologically informative factor in studies of circulating serotonin. Emerging evidence also suggests sexual dimorphism in gut microbiota composition and in microbiota–host signaling pathways, which may contribute to sex differences in circulating serotonin and sleep-related physiology [6, 7]. Sex-associated differences in microbial community structure have been documented across cohorts, with potential consequences for enterochromaffin-cell serotonin biosynthesis and gut–brain communication pathways relevant to sleep regulation [6, 7]. This context motivates prespecified sex-stratified analyses in intervention studies targeting the gut microbiota. The gut microbiota has also emerged as a potential target for sleep modulation. Recent randomized trials in adults have reported probiotic-associated improvements in subjective sleep quality and related well-being outcomes, including studies of *Bifidobacterium breve* CCFM1025 and *Bifidobacterium longum* 1714 in adults with suboptimal sleep or stress-related sleep impairment [8, 9]. At the evidence-synthesis level, recent meta-analyses suggest that probiotics and paraprobiotics may improve some subjective sleep measures, although the magnitude and consistency of effect remain uncertain and objective sleep changes are less clear [10, 11]. Importantly, most of these studies did not measure circulating 5-HT, leaving unresolved whether serotonin-related signaling contributes to the observed sleep-associated effects.

Evidence that probiotics can alter peripheral serotonin in humans is limited and context dependent. In adults with functional constipation, *Lactobacillus reuteri* DSM 17938 reduced circulating serum 5-HT [12], whereas a more recent randomized trial in healthy adults reported that a multi-strain probiotic altered gut-brain-axis-related biomarkers, including plasma serotonin [13]. Together, these findings suggest that probiotic effects on peripheral serotonergic measures may depend on baseline physiology, clinical context, formulation, and the biological matrix analyzed. They also support the rationale for testing whether targeted microbial delivery strategies can influence peripheral 5-HT in otherwise healthy adults.

Microencapsulation is relevant in this context because it is intended to enhance viable probiotic delivery during gastrointestinal transit and may therefore be important when probing gut-derived host responses. Whether this delivery approach modifies peripheral serotonin-related outcomes in the setting of habitual short sleep has not, to our knowledge, been examined. We therefore conducted a pilot randomized, double-blind, placebo-controlled study to examine serum 5-HT in healthy adults receiving microencapsulated *Lacticaseibacillus rhamnosus* GG or placebo for 28 days, with analytical stratification by habitual sleep duration, with exploratory evaluation of sex differences.

## Materials and Methods

**Figure 1:**
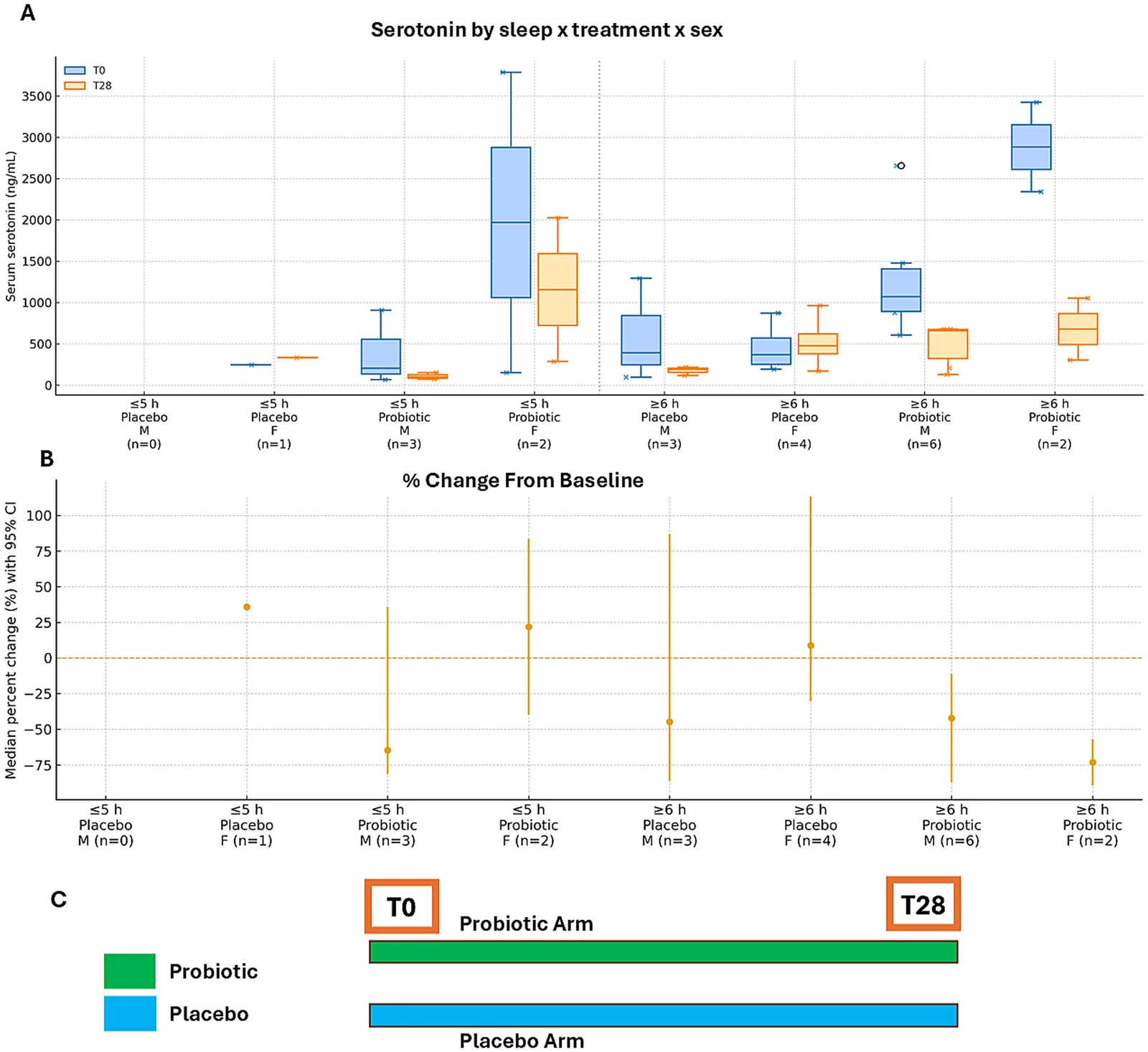
Serum 5-HT by habitual sleep duration, treatment assignment, and sex at baseline (T0) and day 28 (T28), with trial schematic. Boxplots show medians and interquartile ranges; individual values are overlaid. Boxes with *n* ≤ 2 should be interpreted as descriptive summaries only; the full distribution is represented by the individual data points shown. Panel B highlights within-participant change (T28-T0) in the microencapsulated *Lactobacillus rhamnosus* GG group.

We analyzed 21 healthy adults (9 female, 12 male) enrolled in a 28-day interventional study. Baseline participant characteristics by treatment group are summarized in Table 1. Habitual sleep duration was self-reported and was used to classify participants as short sleepers (≤5 h/night, *n* = 6) or adequate sleepers (≥6 h/night, *n* = 15). Habitual sleep duration was assessed at screening using the single question: “On average, how many hours of sleep do you get per night on weeknights?” Participants were randomized to receive microencapsulated *Lacticaseibacillus rhamnosus* GG (1 × 10^10^ CFU/day; *n* = 13) or a matching maltodextrin placebo (5 g/day; *n* = 8) for 28 days using a computer-generated permuted block sequence (block size 4, 2:1 allocation ratio probiotic:placebo), administered by an independent staff member not involved in participant assessments; both participants and investigators were blinded to allocation (Figure 1).

**Table 1:** Baseline participant characteristics by treatment group

Characteristic	Probiotic (*n* = 13)	Placebo (*n* = 8)	Total (*n* = 21)
Age, mean ± SD (years)	29.8 ± 7.4	30.9 ± 6.7	30.2 ± 7.1
Female sex, n (%)	6 (46%)	3 (38%)	9 (43%)
Short sleepers (≤5 h/night), n (%)	5 (38%)	1 (13%)	6 (29%)
Adequate sleepers (≥6 h/night), n (%)	8 (62%)	7 (88%)	15 (71%)

*Age data are mean ± SD. BMI, ethnicity, medication use, and menstrual cycle phase were not collected in this pilot study. Randomization was not stratified by sleep group or sex.*

Fasting venous blood was collected at baseline (T0) and day 28 (T28). Blood was allowed to clot for 1 h at room temperature and centrifuged for 20 min at 1000 × g at 2–8°C. Serum was separated, aliquoted, and stored at -80°C until analysis. Serum 5-HT concentrations were measured by Serotonin ELISA (Novus Biologicals, NBP2-68127). Samples collected at both time points were handled using the same pre-analytical procedure to minimize variability related to platelet activation.

Because serum 5-HT concentrations were markedly right-skewed and sample size was small, values were summarized using medians and interquartile ranges (IQRs), rather than means and standard deviations. Groups were compared using Hodges-Lehmann median differences with Wilcoxon tests. Given sparse strata and a right-skewed distribution, primary inference used a baseline-adjusted ANCOVA of 5% winsorized, log-transformed T28 values, with log-transformed T0 as a covariate and heteroskedasticity-consistent (HC3) robust standard errors. Predictors included treatment, sleep group, sex, and a sex-by-treatment interaction. Serum 5-HT distributions were inspected for extreme observations, and no observations were excluded solely on the basis of their magnitude. Given the marked right-skew and presence of high values, a 5% winsorization strategy followed by log transformation was prespecified to reduce the influence of extreme values in this small sample. We also generated descriptive sex-stratified summaries and sensitivity analyses using nonparametric estimates with bootstrap uncertainty (Table 2; Tables S1–S4). All analyses were performed in R (version 4.3.2; R Core Team, 2023), using the packages sandwich (HC3 covariance estimation), car (Type II ANOVA), and DescTools (winsorization). No formal power calculation was performed because this was an exploratory study. The target of approximately 20 participants was based on recruitment feasibility over the study period and the analyses were intended to generate hypotheses and inform future adequately powered confirmatory studies.

**Table 2:** Median (IQR) 5-HT by stratum and timepoint

SleepGroup	Treatment	Sex	Time	n	Median	Q1	Q3	IQR
≤5 h	Placebo	F	T0	1	245.40	245.40	245.40	-
≤5 h	Probiotic	M	T0	3	203.80	135.00	555.75	420.75
≤5 h	Probiotic	F	T0	2	1968.30	1059.50	2877.10	1817.60
≥6 h	Placebo	M	T0	3	392.50	243.55	842.30	598.75
≥6 h	Placebo	F	T0	4	369.30	250.05	570.08	320.03
≥6 h	Probiotic	M	T0	6	1069.25	892.65	1406.80	514.15
≥6 h	Probiotic	F	T0	2	2881.65	2611.13	3152.17	541.05
≤5 h	Placebo	F	T28	1	333.20	333.20	333.20	-
≤5 h	Probiotic	M	T28	3	97.30	84.65	124.20	39.55
≤5 h	Probiotic	F	T28	2	1155.90	721.40	1590.40	869.00
≥6 h	Placebo	M	T28	3	190.70	153.70	203.80	50.10
≥6 h	Placebo	F	T28	4	477.45	379.93	619.15	239.22
≥6 h	Probiotic	M	T28	6	656.95	320.15	671.18	351.03
≥6 h	Probiotic	F	T28	2	678.10	491.45	864.75	373.30

Single observation; IQR is not calculable and is indicated as -. Values are median (IQR) serum 5-HT (ng/mL). T0, baseline; T28, day 28; M, male; F, female.

All procedures performed in studies involving human participants were in accordance with the ethical standards of the institutional and/or national research committee and with the 1964 Helsinki Declaration and its later amendments or comparable ethical standards. The study protocol was approved by the institutional ethics committee of St. Vincent’s University Hospital Research (protocol No. RS24-063) in March 2024. All participants provided written informed consent before study procedures. The reporting of this exploratory randomized trial followed the applicable CONSORT recommendations.

## Results

Habitual short sleep was not associated with higher serum 5-HT overall (Figure 1; Table S5). At baseline, median 5-HT was 225 ng/mL (IQR 164–742) in short sleepers and 876 ng/mL (IQR 431–1385) in adequate sleepers (pooled Wilcoxon *p* = .205; Figure 1; Table 1; Table S5). The corresponding Hodges-Lehmann median difference was 372 ng/mL, and values in the short-sleep group were highly variable, spanning both the cohort minimum (66 ng/mL) and maximum (3786 ng/mL) at baseline (Figure 1). At T28, median 5-HT values were 219 ng/mL (IQR 111–322) in short sleepers and 450 ng/mL (IQR 199–667) in adequate sleepers (*p* = .267; Figure 1; Table 2; Table S5). The corresponding Hodges-Lehmann difference decreased to 119 ng/mL, consistent with convergence over time (Table S4).

Within the probiotic arm, 5-HT declined in several participants, particularly in those with higher baseline values. For example, one short-sleeping female with an extreme baseline 5-HT concentration (3786 ng/mL) showed a 46% decrease at T28 (Figure 1B; Table S2). This observation was retained in the descriptive analyses, while the influence of extreme values on model-based inference was reduced through winsorization and log transformation. Females also tended to have higher 5-HT than males across strata; at T28, median 5-HT was 450 ng/mL in females versus 199 ng/mL in males (Figure 1; Table S6).

In the baseline-adjusted winsorized-log ANCOVA, there was no evidence of an independent treatment effect (*p* = .33), sex-by-treatment interaction (*p* = .69), or baseline covariate contribution (*p* = .58) (Table 3). The sleep group did not show evidence of an association with T28 5-HT (*p* = .27), consistent with the descriptive analyses. Sex showed evidence of an overall association with T28 5-HT by Type II ANOVA (F = 6.04, *p* = .03; partial η^2^ = 0.29) (Table 4). The model coefficient for female sex corresponded to an estimated ~2.8-fold higher adjusted T28 5-HT, although the coefficient-level *p* value was borderline (β = 1.02 log units; 95% CI 1.00- to 7.77-fold; *p* = .051) (Table 3). Between-arm contrasts within sleep strata were exploratory and not powered for definitive inference (Table S3).

**Table 3:** Baseline-adjusted ANCOVA for T28 serum 5-HT—coefficients (HC3), 95% CI, *p* (untransformed and 5% winsorized-log)

ANCOVA Type	Term	Estimate	95% CI lower	95% CI upper	*p*
Untransformed T28 model	Intercept	85.36	−679.82	850.53	.83
	Treatment[T.Probiotic]	108.07	−556.00	772.15	.75
	Sex[T.F]	339.70	−42.73	722.12	.08
	SleepGroup[T. ≥ 6 h]	−26.46	−1024.90	971.98	.96
	Sex[T.F]:Treatment [T.Probiotic]	−76.68	−998.76	845.40	.87
	T0	0.20	−0.45	0.84	.55
Winsorized log-T28 model	Intercept	3.78	1.69	5.87	<.001
	Treatment[T.Probiotic]	0.58	−0.58	1.75	.33
	Sex[T.F]	1.02	0.00	2.05	.05
	SleepGroup[T. ≥ 6 h]	0.60	−0.46	1.65	.27
	Sex[T.F]:Treatment[T.Probiotic]	−0.29	−1.71	1.13	.69
	logT0	0.13	−0.33	0.58	.58

Baseline-adjusted ANCOVA for T28 serum 5-HT with HC3 robust standard errors. CI, confidence interval.

**Table 4:** Type-II ANOVA for the baseline-adjusted models. F, p, and partial η^2^ for untransformed and 5% winsorized log-transformed models

ANCOVA Type	Term	Sum of squares	df	F	p	partial_eta_sq
ANOVA_TypeII_untransformed_part	Treatment	17610.18	1.00	0.10	0.75	0.01
	Sex	324146.05	1.00	1.93	0.19	0.11
	SleepGroup	2241.07	1.00	0.01	0.91	0.00
	Sex:Treatment	5285.87	1.00	0.03	0.86	0.00
	T0	444056.78	1.00	2.64	0.13	0.15
	Residual	2523489.61	15.00			0.50
ANOVA_TypeII_winsorized_log_par	Treatment	0.55	1.00	1.13	0.30	0.07
	Sex	2.95	1.00	6.04	0.03	0.29
	SleepGroup	0.97	1.00	1.98	0.18	0.12
	Sex:Treatment	0.09	1.00	0.18	0.67	0.01
	logT0	0.23	1.00	0.47	0.50	0.03
	Residual	7.33	15.00			0.50

Type II ANOVA for the baseline-adjusted models. df, degrees of freedom.

## Discussion

In this randomized pilot study, habitual short sleep was not associated with elevated serum 5-HT, in contrast to acute sleep-deprivation studies in which serotonin-related signals increased. These findings suggest that chronic short sleep in free-living adults may not produce a uniform upward shift in peripheral 5-HT and instead may be characterized by substantial inter-individual variability [4]. Within the probiotic arm, serum 5-HT declined in several participants with higher baseline values; however, no independent between-group treatment effect was detected. This pattern likely reflects limited statistical power, and biological variability. The directional declines observed among probiotic-arm participants with high baseline values are consistent with regression to the mean, a phenomenon expected when extreme observations are selected from a distribution with measurement error. In the absence of a statistically significant between-group treatment effect and given the small n, these trends cannot be confidently attributed to a probiotic mechanism rather than to statistical regression. The baseline-adjusted ANCOVA was selected precisely to reduce—though not eliminate—regression-to-the-mean bias by conditioning on T0 values. It is also consistent with prior evidence that probiotic effects on peripheral serotonin are context dependent. For example, *L. reuteri* DSM 17938 reduced serum 5-HT in adults with functional constipation, whereas Walden et al. [13] reported changes in plasma (not serum) serotonin-related biomarkers following a multi-strain probiotic in healthy adults, a matrix distinction relevant to interpretation, as serum and plasma 5-HT concentrations are not directly comparable due to platelet activation during clotting [12, 13]. Recent probiotic trials reporting improvements in sleep quality or well-being did not assess blood 5-HT directly [8, 9], so the relationship between serotonergic shifts and sleep outcomes remains unresolved. At the evidence-synthesis level, recent meta-analyses support modest benefits of probiotics or paraprobiotics on some subjective sleep measures, although objective effects remain less clear [10, 11].

Matrix choice is important when interpreting these findings because serum 5-HT largely reflects platelet-stored serotonin released during clotting, whereas plasma is intended to reflect free circulating 5-HT. Absolute concentrations are therefore not directly comparable across matrices, and pre-analytical platelet activation may add variance even under standardized handling conditions. The higher 5-HT values observed in females are concordant with prior evidence that platelet serotonin uptake sites and serotonergic binding can vary across the menstrual cycle, supporting a role for sex hormones in serotonergic handling [5]. We cannot exclude the possibility that uncontrolled variation in menstrual cycle phase or hormonal contraceptive use among female participants contributed to the observed sex difference and to inter-individual variability in the female subgroup. Future studies should therefore consider timing relative to menstrual cycle phase, hormonal contraceptive use, diurnal sampling, and medication or dietary factors that may influence platelet 5-HT. More broadly, gut microbiota-mediated regulation of enterochromaffin-cell serotonin biosynthesis provides a biologically plausible framework for host serotonergic modulation [1–3], but the extent to which this pathway explains probiotic-linked changes in human peripheral 5-HT remains uncertain.

Taken together, these data provide a pilot evaluation of serum 5-HT in the setting of habitual short sleep during a probiotic intervention and suggest that sex may be an important biological variable. The main limitations are the small sample size, reliance on self-reported sleep duration, and unbalanced strata, all of which restrict inference on subgroup effects. Given the small sample size and resulting limited statistical power, the absence of a detectable treatment effect should not be interpreted as evidence of no effect; adequately powered confirmatory trials are needed. The Sex × Treatment interaction term did not reach statistical significance (*p* = .69), but the study was not powered to detect interaction effects and this analysis remains exploratory. Self-reported sleep duration is known to overestimate objectively measured sleep, and potential misclassification near the ≤5 h/≥6 h boundary may have introduced non-differential bias toward the null for the sleep group comparison. Hormonal status, including menstrual cycle phase and contraceptive use, was not recorded, and these factors may have materially contributed to the higher and more variable 5-HT concentrations observed in female participants. Nonetheless, the sex-related pattern was directionally consistent across descriptive and baseline-adjusted analyses, and participants with higher baseline values showed declines within the probiotic arm. These observations support follow-up studies with adequate power, objective sleep measurement, and prespecified biomarker endpoints. Such trials should test whether probiotic-associated serotonergic modulation is reproducible, whether it varies by sex, and whether formulation-related differences in viable microbial delivery influence peripheral gut-brain biomarkers.

## Supplementary Material

Supplementary_Material_zpag098

## Data Availability

The data underlying this article will be shared on reasonable request to the corresponding author.
