# Supplementary material for: Peripheral sex differences in serum serotonin modulation in habitual short sleepers: an exploratory randomized trial of encapsulated Lacticaseibacillus rhamnosus GG
Source: Sleep Adv. 2026 Sep 2;7(3):zpag098. doi: 10.1093/sleepadvances/zpag098 (PMC13600388; doi:10.1093/sleepadvances/zpag098)
Supplement: Supplementary file 1 [file supplementary_material_zpag098.docx]

**Supplementary material**

**Peripheral sex differences in serum serotonin modulation in habitual short sleepers: an exploratory randomized trial of encapsulated *Lacticaseibacillus rhamnosus* GG**

Gonzalo Saiz-Gonzalo1*, Ahmed W Al-Humadi2, Seamus McSweeney1, Carel W le Roux2,3 and Sinéad B Bleiel1

1	Anabio Technologies, IDA Business Park, Carrigtwohill, Co. Cork, T45 RW24, Ireland

2	Diabetes Complications Research Centre, Conway Institute, University College Dublin, D04V1W8, Ireland

3	Diabetes Research Centre, Ulster University, Coleraine, BT52 1SA, UK

*Corresponding author: Dr Gonzalo Saiz-Gonzalo, Anabio Technologies, IDA Business Park, Carrigtwohill, Co. Cork, T45 RW24, Ireland. Email: gonzalosaiz@anabio.ie

**Supplementary tables**

**Table S1.** 95% bootstrap CIs for stratum medians (T0, T28).

| **SleepGroup** | **Treatment** | **Sex** | **Outcome** | **n** | **Median** | **CI_lo** | **CI_hi** |
| --- | --- | --- | --- | --- | --- | --- | --- |
| ≤5 h | Placebo | M | T0 | 0 |  |  |  |
| ≤5 h | Placebo | M | T28 | 0 |  |  |  |
| ≤5 h | Placebo | F | T0 | 1 | 245.4 | 245.4 | 245.4 |
| ≤5 h | Placebo | F | T28 | 1 | 333.2 | 333.2 | 333.2 |
| ≤5 h | Probiotic | M | T0 | 3 | 203.8 | 66.2 | 907.7 |
| ≤5 h | Probiotic | M | T28 | 3 | 97.3 | 72 | 151.1 |
| ≤5 h | Probiotic | F | T0 | 2 | 1968.3 | 150.7 | 3785.9 |
| ≤5 h | Probiotic | F | T28 | 2 | 1155.9 | 286.9 | 2024.9 |
| ≥6 h | Placebo | M | T0 | 3 | 392.5 | 94.6 | 1292.1 |
| ≥6 h | Placebo | M | T28 | 3 | 190.7 | 116.7 | 216.9 |
| ≥6 h | Placebo | F | T0 | 4 | 369.3 | 193.2 | 871.5 |
| ≥6 h | Placebo | F | T28 | 4 | 477.45 | 169.4 | 962.2 |
| ≥6 h | Probiotic | M | T0 | 6 | 1069.25 | 740.05 | 2066.3 |
| ≥6 h | Probiotic | M | T28 | 6 | 656.95 | 168.75 | 677.4 |
| ≥6 h | Probiotic | F | T0 | 2 | 2881.65 | 2340.6 | 3422.7 |
| ≥6 h | Probiotic | F | T28 | 2 | 678.1 | 304.8 | 1051.4 |

**Table S2.** Within-participant changes (T28-T0): Δ and %Δ by stratum, with medians (IQR) and bootstrap CIs.

| **SleepGroup** | **Treatment** | **Sex** | **Metric** | **n** | **Median** | **Q1** | **Q3** | **IQR** |
| --- | --- | --- | --- | --- | --- | --- | --- | --- |
| ≤5 h | Placebo | M | delta | 0 |  |  |  |  |
| ≤5 h | Placebo | F | delta | 1 | 87.80 | 87.80 | 87.80 | 0.00 |
| ≤5 h | Probiotic | M | delta | 3 | -131.80 | -444.20 | -50.35 | 393.85 |
| ≤5 h | Probiotic | F | delta | 2 | -812.40 | -1286.70 | -338.10 | 948.60 |
| ≥6 h | Placebo | M | delta | 3 | -175.60 | -675.50 | -39.75 | 635.75 |
| ≥6 h | Placebo | F | delta | 4 | 62.95 | 1.50 | 132.25 | 130.75 |
| ≥6 h | Probiotic | M | delta | 6 | -541.50 | -940.05 | -221.47 | 718.58 |
| ≥6 h | Probiotic | F | delta | 2 | -2203.55 | -2660.72 | -1746.37 | 914.35 |
| ≤5 h | Placebo | M | pct_change | 0 |  |  |  |  |
| ≤5 h | Placebo | F | pct_change | 1 | 35.78 | 35.78 | 35.78 | 0.00 |
| ≤5 h | Probiotic | M | pct_change | 3 | -64.67 | -74.01 | -8.85 | 65.17 |
| ≤5 h | Probiotic | F | pct_change | 2 | 21.93 | -12.29 | 56.16 | 68.45 |
| ≥6 h | Placebo | M | pct_change | 3 | -44.74 | -67.85 | 28.42 | 96.28 |
| ≥6 h | Placebo | F | pct_change | 4 | 8.95 | -3.63 | 41.05 | 44.68 |
| ≥6 h | Probiotic | M | pct_change | 6 | -42.12 | -75.44 | -24.71 | 50.74 |
| ≥6 h | Probiotic | F | pct_change | 2 | -73.09 | -82.09 | -64.08 | 18.01 |

**Table S3.** Hodges-Lehmann contrasts: Probiotic - Placebo within each sleep×sex stratum (T0, T28, Δ, %Δ) with CIs and sample sizes.

| **Contrast** | **SleepGroup** | **Sex** | **Outcome** | **HL_diff** | **CI_lo** | **CI_hi** | **n_Probiotic** | **n_Placebo** |
| --- | --- | --- | --- | --- | --- | --- | --- | --- |
| Probiotic − Placebo | ≤5 h | M | T0 |  |  |  | 3 | 0 |
| Probiotic − Placebo | ≤5 h | M | T28 |  |  |  | 3 | 0 |
| Probiotic − Placebo | ≤5 h | M | delta |  |  |  | 3 | 0 |
| Probiotic − Placebo | ≤5 h | M | pct_change |  |  |  | 3 | 0 |
| Probiotic − Placebo | ≤5 h | F | T0 | 1722.90 | -94.70 | 3540.50 | 2 | 1 |
| Probiotic − Placebo | ≤5 h | F | T28 | 822.70 | -46.30 | 1691.70 | 2 | 1 |
| Probiotic − Placebo | ≤5 h | F | delta | -900.20 | -1848.80 | 48.40 | 2 | 1 |
| Probiotic − Placebo | ≤5 h | F | pct_change | -13.85 | -82.29 | 54.60 | 2 | 1 |
| Probiotic − Placebo | ≥6 h | M | T0 | 665.85 | -349.20 | 1382.60 | 6 | 3 |
| Probiotic − Placebo | ≥6 h | M | T28 | 460.50 | 12.60 | 541.20 | 6 | 3 |
| Probiotic − Placebo | ≥6 h | M | delta | -202.90 | -1324.13 | 890.40 | 6 | 3 |
| Probiotic − Placebo | ≥6 h | M | pct_change | -6.72 | -155.61 | 60.74 | 6 | 3 |
| Probiotic − Placebo | ≥6 h | F | T0 | 2349.30 | 1670.05 | 3191.60 | 2 | 4 |
| Probiotic − Placebo | ≥6 h | F | T28 | 112.30 | -428.70 | 741.65 | 2 | 4 |
| Probiotic − Placebo | ≥6 h | F | delta | -2282.20 | -3291.70 | -1257.00 | 2 | 4 |
| Probiotic − Placebo | ≥6 h | F | pct_change | -82.04 | -188.05 | -40.31 | 2 | 4 |

**Table S4.** Hodges-Lehmann contrasts: ≤5 h - ≥6 h within each treatment×sex stratum (T0, T28, Δ, %Δ) with CIs.

| **Contrast** | **Treatment** | **Sex** | **Outcome** | **HL_diff** | **CI_lo** | **CI_hi** | **n_≤5h** | **n_≥6h** | **Wilcoxon_p** |
| --- | --- | --- | --- | --- | --- | --- | --- | --- | --- |
| ≤5 h − ≥6 h | Placebo | M | T0 |  |  |  | 0 | 3 | NA |
| ≤5 h − ≥6 h | Placebo | M | T28 |  |  |  | 0 | 3 | NA |
| ≤5 h − ≥6 h | Placebo | F | T0 | -123.9 | -626.1 | 52.2 | 1 | 4 | 0.8 |
| ≤5 h − ≥6 h | Placebo | F | T28 | -144.25 | -629 | 163.8 | 1 | 4 | 0.8 |
| ≤5 h − ≥6 h | Probiotic | M | T0 | -774.4 | -1579.35 | -35.2 | 3 | 6 | 0.095 |
| ≤5 h − ≥6 h | Probiotic | M | T28 | -526.3 | -585.9 | -57.3 | 3 | 6 | 0.0476 |
| ≤5 h − ≥6 h | Probiotic | F | T0 | -913.35 | -3272 | 1445.3 | 2 | 2 | 1 |
| ≤5 h − ≥6 h | Probiotic | F | T28 | 477.8 | -764.5 | 1720.1 | 2 | 2 | 1 |

**Table S5**. Pooled Wilcoxon tests (≤5 h vs ≥6 h across all participants)

| **Outcome** | **n_≤5h** | **n_≥6h** | **Wilcoxon_p (pooled)** |
| --- | --- | --- | --- |
| T0 | 6 | 15 | 0.2051 |
| T28 | 6 | 15 | 0.2672 |

**Table S6.** Hodges-Lehmann contrasts: Female - Male within each sleep×treatment stratum (T0, T28, Δ, %Δ) with CIs.

| **Contrast** | **SleepGroup** | **Treatment** | **Outcome** | **HL_diff** | **CI_lo** | **CI_hi** | **n_F** | **n_M** |
| --- | --- | --- | --- | --- | --- | --- | --- | --- |
| Female − Male | ≤5 h | Placebo | T0 |  |  |  | 1 | 0 |
| Female − Male | ≤5 h | Placebo | T28 |  |  |  | 1 | 0 |
| Female − Male | ≤5 h | Placebo | delta |  |  |  | 1 | 0 |
| Female − Male | ≤5 h | Placebo | pct_change |  |  |  | 1 | 0 |
| Female − Male | ≤5 h | Probiotic | T0 | 1481.35 | -757.00 | 3719.70 | 2 | 3 |
| Female − Male | ≤5 h | Probiotic | T28 | 1044.35 | 135.80 | 1952.90 | 2 | 3 |
| Female − Male | ≤5 h | Probiotic | delta | -449.65 | -1792.10 | 892.80 | 2 | 3 |
| Female − Male | ≤5 h | Probiotic | pct_change | 40.12 | -93.49 | 173.73 | 2 | 3 |
| Female − Male | ≥6 h | Placebo | T0 | -23.20 | -1023.10 | 479.00 | 4 | 3 |
| Female − Male | ≥6 h | Placebo | T28 | 301.00 | 2.60 | 758.40 | 4 | 3 |
| Female − Male | ≥6 h | Placebo | delta | 238.55 | -60.90 | 1266.10 | 4 | 3 |
| Female − Male | ≥6 h | Placebo | pct_change | 53.09 | -94.09 | 161.20 | 4 | 3 |
| Female − Male | ≥6 h | Probiotic | T0 | 1600.55 | 814.15 | 2546.80 | 2 | 6 |
| Female − Male | ≥6 h | Probiotic | T28 | 273.85 | -370.80 | 843.20 | 2 | 6 |
| Female − Male | ≥6 h | Probiotic | delta | -1214.95 | -2917.60 | -301.80 | 2 | 6 |
| Female − Male | ≥6 h | Probiotic | pct_change | -28.53 | -68.23 | 27.51 | 2 | 6 |
